# Factors Determining the Implementation of Measures Aimed at Preventing Zoonotic Diseases in Veterinary Practices

**DOI:** 10.3390/pathogens10040436

**Published:** 2021-04-06

**Authors:** Véronique Renault, Sébastien Fontaine, Claude Saegerman

**Affiliations:** 1 Research Unit in Epidemiology and Risk Analysis Applied to Veterinary Sciences (UREAR-ULiege), Fundamental and Applied Research for Animal Health (FARAH) Centre, Faculty of Veterinary Medicine, University of Liege, 4000 Liège, Belgium; vrenault@uliege.be; 2 Research Institute in Social Sciences, Department of Social Sciences, Faculty of Social Sciences, University of Liege, 4000 Liège, Belgium; Sebastien.Fontaine@uliege.be

**Keywords:** one health, biosecurity, veterinarians, students, Health Belief Model, perception, risk, behaviour

## Abstract

Background: Zoonoses prevention relies mainly on the implementation of different biosecurity measures. This study aimed to assess the level of implementation of biosecurity measures by veterinary practitioners and students and to identify the possible behaviour change determinants. Methods: The data was collected through a cross-sectional survey (N = 382). Statistical analyses were implemented based on the Health Belief Model to identify the possible determinant of the behaviours and the explanatory variables of the perceptions. Results: The survey showed a good level of implementation of the biosecurity measures (median of 81%). The implementation was associated with a higher perception of the zoonoses’ susceptibility and the measures’ benefits, and with a lower perception of the zoonoses’ severity. The study also revealed that the decision to implement a measure was mainly taken on a case-by-case basis depending on the perceived risk of exposure related to a specific context or intervention. Conclusion: The main determining factors identified for the implementation of biosecurity measures (BSMs) were the risk susceptibility and the benefits of the biosecurity measures, which could be influenced by evidence-based communication. The methodology developed can be applied regularly and in other countries to better capture these changes in perceptions over time.

## 1. Introduction

Biosecurity (BS) includes all the measures aiming at preventing the introduction of pathogen agents and/or reducing their transmission. As part of the “One Health” approach, BS is particularly important as it includes measures preventing animal, human and environmental contaminations. As more than 75% of emerging diseases and 60% of the infectious diseases affecting humans are zoonotic [1], BS in public health needs to address the issue of animal–human contaminations. In that regard, veterinarians represent a population at greater risk of infection by zoonotic pathogens and can play a role in their transmission [2]. Despite this accrued risk, the level of implementation of biosecurity measures (BSMs) by veterinarians was reported as generally low in several studies [3,4,5,6,7,8,9,10]. Therefore, it would be worth investigating the reasons for this low implementation despite the risks.

**Figure 1:**
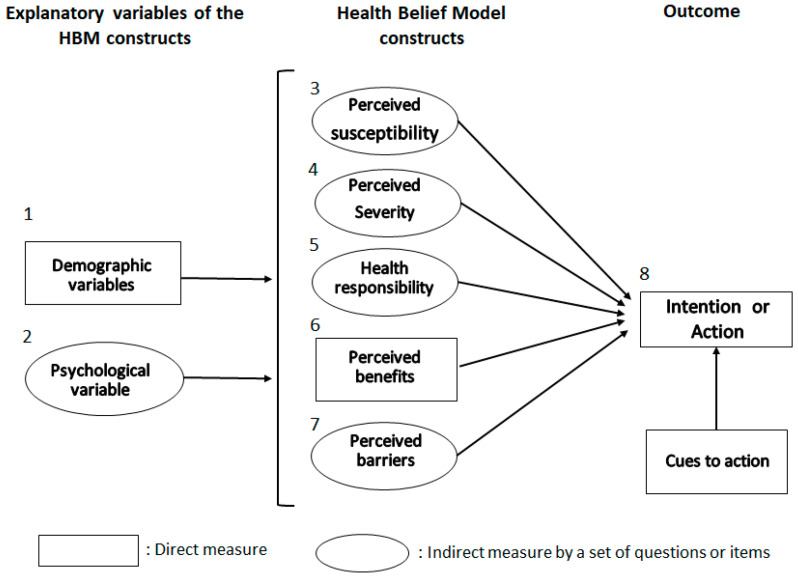
The Health Belief Model.

According to the Health Belief Model (HBM), health-related behaviours are influenced by different beliefs and perceptions which can be influenced by different psychosocial determinants, as described in Figure 1 [11]. The perceptions listed, also called HBM constructs, are: (i) the risk susceptibility (perceived likelihood of the risk of occurrence), (ii) the risk severity (perceived impact of the risk if it occurs), (iii) the benefits (perceived positive outcomes related to a given behaviour), (iv) the barriers (perceived barriers to the behaviour implementation or outcomes) and (v) the health responsibility (perceived responsibility towards animal, public and environmental health). The determinants of good behaviours must be identified to better promote the necessary changes and to better mitigate the zoonotic risks. Nevertheless, such studies have not yet been conducted for the veterinary profession.

The objective of this study was to assess the level of implementation of BSMs by veterinary practitioners and senior students in veterinary medicine (years 5 and 6 of the veterinary course at Liege) and to identify the main determinants of the adoption of BSMs. The outcomes of the study make it possible to better communicate with veterinarians and to increase the implementation level of BSMs in their practices.

## 2. Results

### 2.1. Survey Results and Respondents Profiles

The answer rates were 35% for the students (N = 227) and 13% for the veterinary practitioners (N = 114) (Table 1).

The proportion of males and females among the student respondents was 22% and 78% respectively and a large majority (93%) reported having practical experience through various internships. The veterinary practitioners were 51% male and 49% female.

Among the respondents, 52% were practicing on or had experience with small animals only, 10% with large animals only, 32% had mixed practices (including equine) and 6% had no practical experience (students) or were practicing in other fields, such as wildlife and consultancies (Table 1). Fifteen per cent of the students and 39% of the veterinary practitioners who answered the survey reported having been personally affected by a zoonosis in the past. Thirty per cent of the students and 71% of the practitioners reported knowing someone that had been affected by a zoonosis in the past.

The chi-square tests to compare the proportion of men and women and the proportion of veterinarians in the different types of practices did not demonstrate any statistical differences (*p* > 0.05). We can therefore consider the samples as representatives of the overall population.

### 2.2. Implementation Level of the Biosecurity Measures

**Figure 2:**
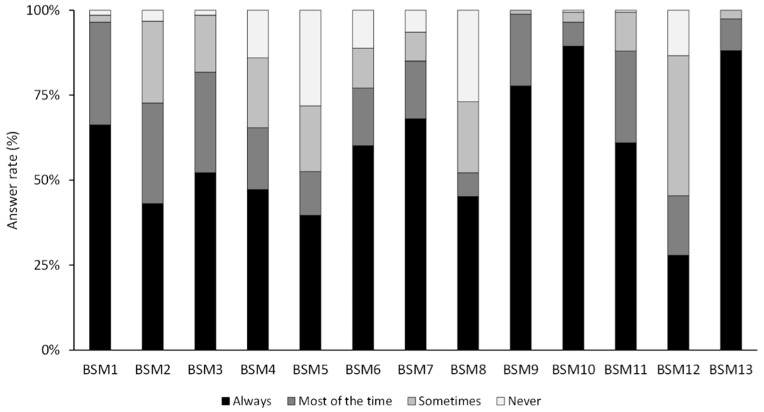
Implementation level of the different biosecurity measures by the respondents (N = 341). Legend: biosecurity measure 1 (BSM1): hands disinfection; BSM2: asking about the country of origin of the animal; BSM3: wearing gloves adapted to needs; BSM4: wearing a mask in case of interventions likely to cause projections; BSM5: wearing protective goggles during interventions likely to cause projections; BSM6: throwing the needles directly into a specific container without replacing the cap; BSM7: washing dirty clothing separately with a proper cleaning cycle; BSM8: being vaccinated against rabies; BSM9: ensuring a proper containment; BSM10: proceeding to an immediate disinfection if dealing with a wounded animal; BSM11: following updates through continuous training; BSM12: using a disposable coat; BSM13: cleaning boots when exiting the holdings.

As reported by the respondents, most of the BSMs were implemented either always or most of the time (Figure 2). Overall, the BS score reflecting the percentage of implementation of the different biosecurity measures ranged from 46% to 100%, with a median of 81% (quartile 1: 69%, and quartile 3: 92%). Some measures—such as disinfecting the hands after each consultation, ensuring proper containment of the animals, proceeding to an immediate disinfection if dealing with a wounded animal and, for rural practitioners, cleaning boots when exiting a holding—were always implemented or implemented most of the time by more than 95% of the respondents. Three BSMs had a lower implementation rate (45–53%): (i) wearing protective glasses, (ii) being vaccinated against rabies and (iii) using disposable coats (for rural practitioners).

The conditions for a measure to be implemented (when not done systematically) or adopted (when a measure was reported as never implemented) varied among the respondents (Table 2). Nevertheless, overall as well as for the large majority of the BSMs (8 out of 13), risk-based decisions or the low perception of the risk exposure was the main reason justifying non-implementation.

### 2.3. Assessment of the Reliability of the Items Used to Indirectly Determine the Psychological Variable and the Health Belief Model Constructs

The Cronbach’s alpha standardised coefficient calculated for the “risk aversion” as well as the perception of “risk susceptibility” and the “health motivation” showed a good reliability (coefficient > 0.7) while the reliability of “risk severity” and “barriers” seemed poor (coefficient = 0.52 and 0.53, respectively) (Table 3). The exploratory factor analysis (EFA) nevertheless confirmed “barriers” as a factor build based on three items despite this poor Cronbach’s alpha coefficient. The final scores of each construct were therefore calculated considering the items providing the highest Cronbach’s alpha (Table 3).

**Figure 3:**
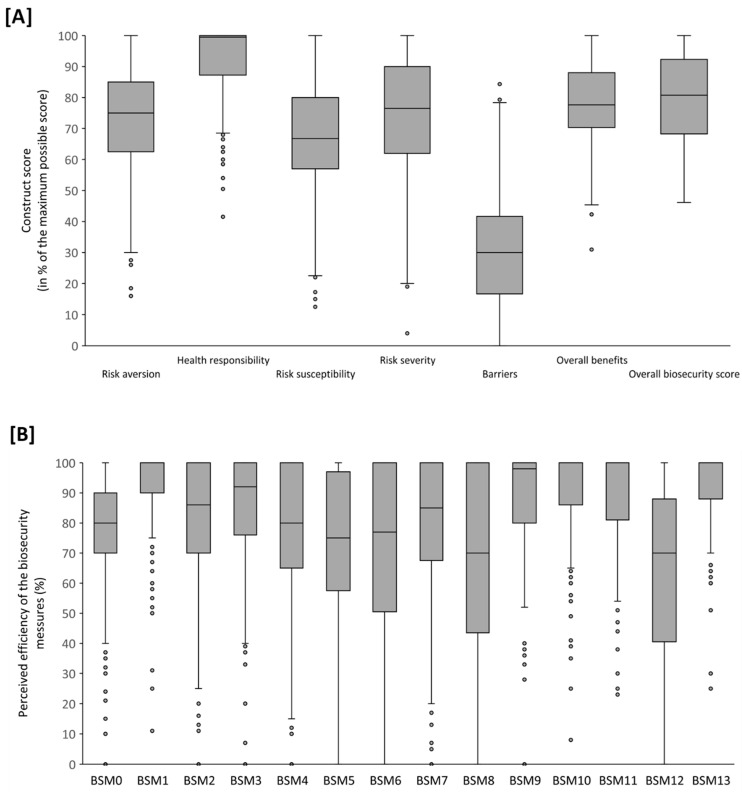
Perceptions of the different Health Belief Model constructs (**A**) and of the perceived benefits of the specific biosecurity measures (**B**). Legend: BSM0: overall efficiency of the different preventive measures; BSM1: hands disinfection; BSM2: asking about the country of origin; BSM3: wearing gloves adapted to needs; BSM4: wearing a mask (if there is a risk of projections); BSM5: wearing protective goggles (if there is a risk of projections); BSM6: throwing the needles away directly without replacing the cap; BSM7: washing dirty clothing separately; BSM8: being vaccinated against rabies; BSM9: ensuring a proper containment; BSM10: proceeding to an immediate disinfection if dealing with a wounded animal; BSM11: attending continuous training; BSM12: using a disposable coat; BSM13: cleaning boots when exiting the holdings.

The median scores obtained for the psychological variable and the different HBM constructs were generally high, with the median values being above 67%, with the exception of the scores for perceived “barriers”, which were generally low, with a median value of 30% (Figure 3).

### 2.4. Regression Models

The Spearman rank correlation test and the Kruskal–Wallis H Test showed no significant associations between the different explanatory variables of the HBM constructs.

**Figure 4:**
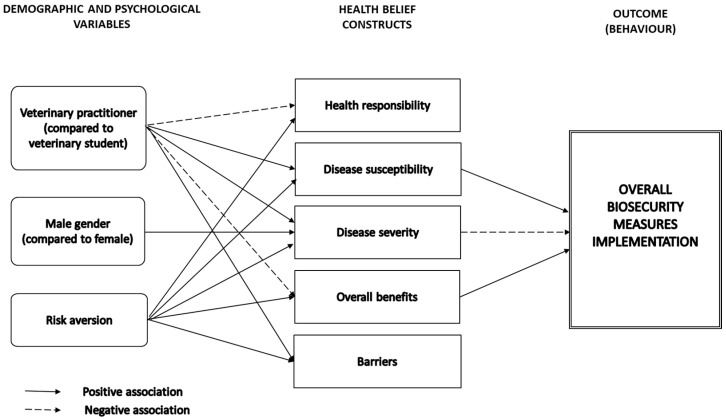
Identification of significant associations based on the multivariable regression model using the overall benefits perception and the overall biosecurity score (for the veterinary practitioners and students).

When considering the overall BSM implementation score as the final output and the overall BSM benefits perception, the final multivariable model showed that the overall BS implementation was significantly and positively associated with the perception of the disease susceptibility and the perceived benefits, while it was negatively and significantly associated with the perception of the disease severity (Figure 4 and Appendix B). The HBM constructs were significantly associated with three explanatory variables (Figure 4). The seniority of the veterinary practitioner, type of practice, risk aversion profile and workload had no significant effects on the different perceptions. A high-risk aversion profile was associated with a higher perception of the five HBM constructs, and the perception of the zoonoses’ severity was significantly higher for males. The veterinary practitioners had a significantly higher perception of the diseases’ severity and barriers while they had a lower perception of their health responsibility and of the overall benefits of the BSM implementation regarding the prevention of zoonoses.

**Figure 5:**
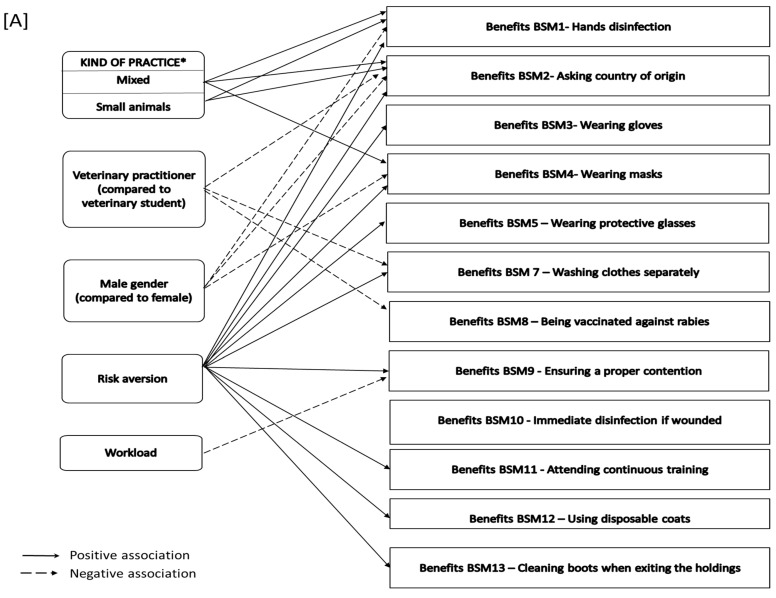

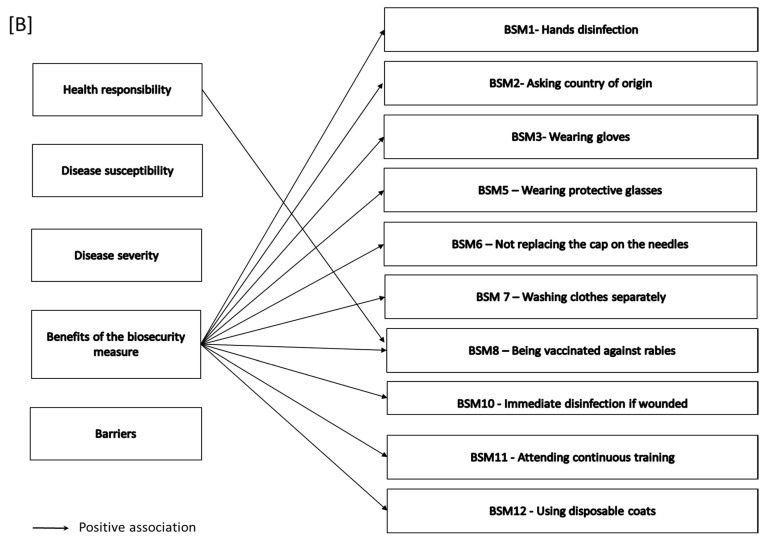
Identification of significant associations identified based on the multivariable regression model for the different biosecurity measures (for veterinary practitioners and students). (**A**) Variables significantly associated with the perceived benefits of the biosecurity measures and (**B**) HBM constructs significantly associated with the implementation of the different biosecurity measures. Legend: * mixed and small animal practices compared to large animal practices.

Regression models were also developed for each of the specific BSMs listed in the survey relating to the factors affecting the perception of their benefits (Figure 5A) and their actual implementation (Figure 5B). With regard to the perceived benefits of the different BSMs, and with the exception of BSM6 (throwing away needles without replacing the cap), respondents with a higher “risk aversion” level had a significantly higher perception of the benefits. Male respondents had a significantly lower perception of the benefits of hands disinfection, asking about the country of origin and wearing masks. Compared to veterinary students, veterinary practitioners had a significantly lower perception of the following BSMs: asking about the country of origin, washing working clothes separately and being vaccinated against rabies. The type of practice also seemed to significantly influence the perceptions of some BSM benefits. Compared to rural practitioners, the veterinarians working with small animals (or in mixed practices) seemed to have a higher perception of the benefits of hands disinfection, asking about the country of origin and wearing masks. The workload seemed to negatively influence the perceived benefits of ensuring proper containment of the animals.

With regard to the factors determining the BSMs’ implementation, the perception of the benefits was significantly and positively associated with the implementation level of all the BSMs, with the exception of three (wearing a mask, ensuring proper containment and cleaning boots when exiting an animal holding) for which no association was found with any of the HBM constructs. Respondents with higher “health responsibility“ were also more likely to be vaccinated against rabies.

## 3. Discussion

This is the first study evaluating the level of implementation of BSMs by Belgian veterinarians and veterinary students with regard to zoonosis prevention. It also identified the possible influence of personal beliefs and perceptions on the adoption of BSMs, as well as possible cues to action, in order to influence the decision-making process. To reduce the volunteer and the social desirability biases the anonymity of the respondent was guaranteed, the questions were oriented around the respondents’ daily practices and several reminders were sent in order to increase the response rate. The answer rates seemed to be acceptable, as it was more efficient than the reported response rate of 4.7% for personalised internet surveys [12]. The samples were considered representative of the overall population, as the proportion of males and females as well as the proportions of different type of practices were not significantly different among the target populations and the respondents.

The reported level of implementation of the BSMs by the veterinary practitioners was generally good, with 8 out of the 13 BSMs being systematically implemented by more than 50% of the respondents. If this situation is comforting, it seems in conflict with the results of previous studies mentioning lower implementation rates, including in a Belgian study targeting rural practitioners [8]. These differences could be explained by the difference in the respondents’ profiles (the previous study was addressed to rural practitioners only) or differences in the formulation of the questions, which might lead to different answers. It is therefore difficult to compare the outcomes of the different studies.

For most BSMs, and overall, the main reason for non-implementation was the perceived low exposure to the risk. The second most listed reason, “relevance of the measure based on the type of practice or intervention”, can also be assimilated to a perceived exposure to the risk, which is considered higher or lower based on the kind of practice or intervention and determines the decision to implement a given BSM or not. This means that most veterinarians will decide to implement a given BSM on a case-by-case basis, which requires going through a systematic risk analysis. This is in line with the findings of other studies [5,6] and represents a major concern as this risk analysis is mainly based on individual perceptions and might not reflect the actual level of risk. An ecological concern appears to prevent the implementation of two BSMs: washing clothes separately and wearing disposable coats. The negative ecological impact of these BSM is perceived by the respondents as more important than the actual risk of being infected by a zoonosis. This concern should be addressed by finding some efficient, adapted and ecological friendly solutions and properly considered in any communication messages.

The HBM constructs were significantly different among the student and veterinarian populations. The fact that veterinary students had a higher perception of their health responsibility and of the overall benefits of the BSM and a lower perception of zoonoses’ susceptibility and severity, as well as a lower perception of the barriers, is an interesting finding which would be worth investigating further. It might be related to personal experiences with zoonoses, as the percentage of respondents who were affected by a zoonosis in the past was higher for the practitioners compared to the veterinary students (39% and 15% respectively). Small animal practitioners and mixed practitioners had a significantly higher perception of the benefits of several BSMs: hands disinfection, asking about the country of origin and wearing masks. The lower perception of the benefits of asking about the country of origin (for rabies prevention) was lower in the rural practitioners; it can be explained by the lower risk of exposure for veterinarians not working with carnivores. For hands washing and wearing a mask, the lower perception of the measures’ benefits could not be associated with any logical explanation, although it appears from the comments that several rural practitioners mentioned that wearing a mask was not well-received by farmers and that water facilities were not always available on the field. Therefore, the perceptions of the benefits for these two measures might have been lowered by these inconveniences being perceived as important and giving a “negative balance” to the benefits.

The overall biosecurity measures implementation was significantly higher for the respondents with a higher perception of susceptibility to zoonoses and of BSM benefits. This is in line with the analysis of the reasons for non-implementation of the measures, for which the majority were based on a perceived low risk of exposure, in the cases of respondents never implementing a measure, or, in cases of respondents only implementing a measure in some cases, a higher perception of risk (e.g., suspicion of a zoonosis). The implementation level was also significantly lower when the perception of the severity of the zoonoses was higher, which is surprising as we assumed that the perception of the zoonoses’ severity would positively influence the behaviour implementation. Indeed, a previous meta-analysis based on vaccination examples proved “the consistent relationships between risk perceptions and behaviour” and considered risk perceptions as a key concept in different theories of health behaviour [13]. The perceptions of barriers did not have a significant influence on the studied behaviour but other studies based on the HBM identified barriers as a significant component [14,15]. This difference could be linked to what were defined as “barriers” in the different studies. In the present study, “barriers” were defined as the perceived level of control over the diseases while, in some other studies, barriers were defined as constraints, such as the cost or burden of the measure. The main limitation of the cross-sectional studies applied to behaviour change analysis is that perceptions might also be influenced by existing behaviour (e.g., a respondent vaccinated against rabies might have a lower perception of the susceptibility to infection than a non-vaccinated respondent) [11]. This is why the best approach would be a prospective interventional study where the behaviour changes are evaluated instead of the actual behaviours and compared between a control group and a group who benefitted from interventions [11]. These kinds of experimental studies have been reviewed in a meta-analysis [16] which concluded that “the impact of risk appraisals on behaviour is moderated by efficacy appraisals”. Therefore, the risk appraisal, which includes the risk susceptibility and severity, generally has a significant effect on the behaviour change but this effect is mitigated by the efficacy approval, defined as “people’s judgment of their ability to manage a focal hazard” based on the efficacy of the possible measures or behaviours. The logic behind this finding is that, if an individual has a high perception of the efficacy of a preventive measure, they will be more likely to adopt the behaviour when their risk perception increases, and, if the individual believes they have no control over the risk (low efficacy approval), the risk perception will not affect the behaviour change.

## 4. Materials and Methods

### 4.1. Survey Design and Implementation

The data were collected in two online surveys developed with LimeSurvey, an open-source web application. One questionnaire targeted the veterinary practitioners in Wallonia (Appendix C) and the other was directed to the veterinary students of the University of Liege in the second or third years of the Master’s degree (Appendix D). The study was based on the HBM and different questions were asked to assess HBM constructs and the level of implementation of the BSMs by the respondent.

The demographic variables considered in the survey were gender, the year of education or the year of graduation, the type of practice (large animals, equine, small animals, mixed or other) and the workload. One psychological variable, “risk aversion”, was assessed indirectly by asking the respondents their degree of agreement (from 0: fully disagree to 100: fully agree) to three different statements, formulated as questions provided in the form a validated risk attitude scale [17].

The questions used to assess the five HBM constructs were formulated based on existing guidelines [18,19] and questionnaires used in previous studies [20,21,22,23,24]. The constructs were assessed indirectly by asking the respondents their degree of agreement (from 0: fully disagree to 100: fully agree) to different statements (Table 4), with the exception of the perceived benefits of the different BSMs, which were assessed through a direct question (Table 2). The risk of infection by a zoonotic pathogen was assessed in terms of susceptibility (perceived likelihood to occur) and severity (perceived impact of the risk if it occurs). For “benefits”, the perceived efficiency of the BSM implementation regarding the prevention of zoonoses was assessed both globally and individually for the 13 BSMs listed as good practices in veterinary medicine [25]. The “barriers” were defined as the perceived level of control the respondent had on the risk management measures and their ability to perform them. The last construct, “health responsibility”, referred to the sense of responsibility perceived by the respondent regarding their health, public health and animal health.

The last component of the HBM model is “intention or action”. For the veterinary students, the questions asked whether their intention would be to perform the BSM in their future practice, while for the veterinary practitioners, the questions asked if they applied the BSM in their daily practice. In both cases, the respondents were asked if the BSM would be implemented, or was implemented: always, most of the time, sometimes or never. The question was asked for each of the 13 BSMs used to measure the “benefit” construct (Table 1). “Cues to action” are defined in the HBM model as a “stimulus necessary to trigger the decision-making process” [14]. These elements are various and could not be clearly defined prior to the survey. In order to identify the possible factors that could trigger the decision-making process, whenever a respondent stated that a BSM was not always implemented, they were asked: (i) in which specific circumstances they were implementing the BSM and (ii) the main reason for not implementing the BSM (if they perceived it to be efficient but reported not implementing it).

Before validation, the questionnaires were pre-tested by four veterinarians (two rural practitioners and two small animal practitioners) and six veterinary students, respectively. Invitations to answer the survey were sent to the students through the mailing lists of the Students’ Office and to the veterinary practitioners by the Professional Union of Veterinarians in Wallonia. The questionnaire was available from 15 September 2019 to 15 May 2020, with monthly reminders sent between 26 September 2019 and April 2020.

### 4.2. Statistical Analysis

The data from the completed questionnaires were extracted to Microsoft Excel© and the responses given by the participants were coded in accordance with Appendix A.

The representativeness of the samples was tested with a chi-square test performed in Stata SE/14 (StataCorp, College Station, TX, USA) by comparing the proportion of men and women in both populations and, for the veterinary practitioners, the representation of the different type of practices in both groups.

### 4.3. Scoring of the Health Belief Model Components

The psychological variable “risk aversion” and the four HBM constructs were determined indirectly through a set of questions or items. The items to be included in the construction of the construct were confirmed with an exploratory factor analysis performed with the lavaan package in R studio© (version 3.6.1 2019-07-05) and the Cronbach’s alpha coefficient (α) using the psych package in R studio© (version 3.6.1 2019-07-05). For the EFA, items with a factor loading equal or superior to 0.3 [26] were considered as important, a Cronbach’s alpha coefficient equal to or above 0.7 was considered to demonstrate a good reliability and coefficients above 0.6 were considered to demonstrate an acceptable reliability [27]. For each construct, a mean score ranging from 0 to 100 was calculated after the identification of the items to be included. The scores of the reverse-worded questions in which the component was negatively formulated were recalculated to ensure uniformity across questions and facilitate the analysis (a higher score therefore always represented a higher perception of the construct measured).

The other components of the HBM, the perceived benefits and the intention or action, were assessed directly with a single question. The perceived benefits were assessed through an efficiency score ranging from 0 to 100. An overall score for the perception of benefits was also determined by calculating the average score of all the BSM benefits (13 in total). In terms of intention or action, an overall BS score was calculated and expressed as a percentage of the maximum score possible.

(1) $$\mathrm{Overall}\;\mathrm{BS}\;\mathrm{score}=\frac{\sum_{x=1}^{13}\mathrm{Implementation}\;\mathrm{level}\;\mathrm{of}\;\mathrm{BSM}\left(x\right)}{\sum_{x=1}^{13}\mathrm{Maximum}\;\mathrm{score}\;\mathrm{of}\;\mathrm{BSM}\left(x\right)}\times 100$$

### 4.4. Negative Binomial Regression Models

In order to identify the main determinants of the adoption of BSMs, different multivariable regression models were used in order to assess: (i) the influence of the different demographic and psychological variables (explanatory variables) on each of the HBM constructs (outcomes) and (ii) the influence of the different HBM constructs (explanatory variables) on the “intention or action” (outcome). The HBM constructs and the “BS score” were considered as a count ranging from 0 to 100. A Poisson regression was therefore used initially but the goodness of fit of the Poisson regression appeared insufficient due to extra-binomial variability. A negative binomial regression was therefore used for the different analyses.

Prior to the model testing, possible correlations between the exploratory variables to be used were tested using a Spearman rank correlation test for the continuous variables and a Kruskal–Wallis H Test for comparisons between continuous and categorical variables. At first, univariable negative binomial regressions were implemented to assess the possible effect of the explanatory variables on the outcome variable. All the explanatory variables for which a significant difference was identified (*p*-value < 0.1, in order to be more conservative) were included in the multivariable negative binomial regression model. A backwards stepwise procedure was then applied in Stata SE/14 (StataCorp LP, College Station, TX, USA). The model was progressively simplified by removing the less significant variables with a *p*-value > 0.05 one by one. The model was considered as final when all variables had a significant *p*-value (<0.05), or when no further simplification was possible without having a significant difference between the most complex and the simpler model (likelihood ratio test with a *p*-value < 0.05).

## 5. Conclusions

Based on the findings of this study, it appears that the main factors that can positively influence the actual implementation of BSMs are the perception of the risk susceptibility and the perception of the BSM benefits or their relevance. In order to facilitate this implementation it would be necessary to deploy a different kind of evidence-based study, which could support the different communication message and convince the veterinarians of the relevance and efficacy of the measure. Nevertheless, due to the complexity of the interrelations between the different beliefs, perceptions and the behaviours and their specificity to a given context, the outcomes of this study should not be generalised to other countries and might change over time based on the national context. Prospective and observational studies assessing the evolution and duration of all these elements over time might help better predict and influence behaviours in a broader context, as well as increase the efficiency of the awareness raising campaigns.

## Figures and Tables

**Table 1:** Demographics of the respondents.

Responders	Year of Study or Graduation	N	Gender		Kind of Practice			
			Female	Male	None/Other	Large Animals	Small Animals	Mixed
Veterinary students	Total students	960	76.35%	23.65%	-	-	-	-
	Total respondents	227	78.41%	21.59%	7.49%	3.96%	48.90%	39.65%
	2nd year Master’s	162	75.31%	24.69%	9.88%	3.09%	50.00%	37.04%
	3rd year Master’s	65	86.15%	13.85%	1.54%	6.15%	46.15%	46.15%
Veterinary practitioners	Total UPV members solicited	848	38.92%	61.08%	0.00%	11.79%	26.42%	61.79%
	Total respondents	114	49.12%	50.88%	3.51%	21.05%	58.77%	16.67%
	Before 1986	28	3.57%	96.43%	7.14%	17.86%	57.14%	17.86%
	1986 to 1995	38	52.63%	47.37%	5.26%	23.68%	57.89%	13.16%
	1996 to 2005	25	68.00%	32.00%	0.00%	20.00%	68.00%	12.00%
	2006 to now	23	78.26%	21.74%	0.00%	21.74%	52.17%	26.09%
TOTAL		341	68.62%	31.38%	6.16%	9.68%	52.20%	31.96%

**Table 2:** List of most frequent conditions under which a given biosecurity measure was or would be implemented (N = 341).

Condition of Implementation	OVERALL	BSM	BSM	BSM	BSM	BSM	BSM	BSM	BSM	BSM	BSM	BSM	BSM	BSM
		1	2	3	4	5	6	7	8	9	10	11	12	13
Numbers of answers	1059	96	127	62	123	143	74	78	78	43	17	72	127	19
Risk-based (increased risk/evidence-based risk)	**37%**	**34%**	**77%**	**56%**	**34%**	**29%**	**11%**	**27%**	**62%**	**14%**	**24%**	**1%**	**42%**	**11%**
Relevance	**10%**	0%	1%	2%	**23%**	**15%**	3%	5%	**13%**	0%	0%	0%	**29%**	0%
Materials (or infrastructure) availability	**7%**	4%	0%	3%	2%	4%	**30%**	1%	1%	2%	**24%**	**26%**	1%	**79%**
Feasibility	7%	**19%**	0%	0%	**9%**	8%	3%	6%	3%	**53%**	6%	1%	0%	0%
Not specified/I do not know	7%	**23%**	6%	6%	7%	3%	7%	4%	3%	2%	12%	6%	4%	**5%**
Sufficient time	5%	10%	1%	**8%**	2%	3%	1%	4%	0%	5%	**35%**	**31%**	0%	0%
More practical/comfortable	5%	3%	0%	**8%**	7%	**22%**	1%	1%	1%	0%	0%	1%	0%	0%
More discipline (negligence)	5%	6%	**7%**	2%	**9%**	7%	5%	5%	**5%**	2%	0%	0%	0%	0%
Recyclable (ecological concern)	3%	0%	0%	2%	0%	0%	0%	**17%**	0%	0%	0%	0%	**18%**	0%
Knowledge/information	3%	0%	0%	0%	1%	0%	**22%**	1%	0%	0%	0%	**19%**	0%	0%
Financial sustainability and/or justification	2%	0%	0%	6%	0%	1%	0%	**18%**	0%	0%	0%	6%	2%	0%
Willingness to do it	2%	0%	0%	0%	0%	5%	**16%**	1%	**5%**	0%	0%	0%	1%	0%
Good acceptance/usual practice	2%	0%	**7%**	5%	6%	2%	0%	0%	0%	0%	0%	0%	0%	0%
Proven to be efficient/needed	2%	0%	0%	0%	1%	0%	0%	4%	1%	**21%**	0%	1%	1%	**5%**

Legend: grey cells: most frequent reasons; bold numbers: among the three most listed reasons. BSM1: hands disinfection; BSM2: asking about the country of origin of the animal; BSM3: wearing gloves adapted to needs; BSM4: wearing a mask (if there is a risk of projections); BSM5: wearing protective goggles (if there is a risk of projections); BM6: throwing away the needles without replacing the cap; BM7: washing dirty clothing separately; BM8: being vaccinated against rabies; BM9: ensuring proper containment; BM10: proceeding to an immediate disinfection if dealing with a wounded animal; BM11: attending continuous training; BM12: using a disposable coat; BM13: cleaning boots when exiting the holdings.

**Table 3:** Cronbach’s alpha coefficient, factor loadings and scoring formulas used to determine the scores of the respective components.

Component	Cronbach’s α	Item Code	α if Item Deleted	Factor Loadings (EFA Analysis)	Component Score Calculation
Risk aversion	0.8	RA1	0.6	0.8	Risk aversion score = (RA1 + RA2)/2
		RA2	0.6	0.9	
		RA3	0.8	0.5	
Risk susceptibility	0.8	Su1	0.8	0.6	Risk susceptibility score = SUM Su1 to Su4/4
		Su2	0.7	0.8	
		Su3	0.7	0.7	
		Su4	0.8	0.6	
Risk severity	0.6	Se1	0.4	0.7	Risk severity score = (Se1 + Se2)/2
		Se2	0.2	0.8	
		Se3	0.7	0.0	
Health responsibility	0.7	HR1	0.5	0.8	Health responsibility score = (HR1 + HR2)/2
		HR2	0.5	0.9	
		HR3	0.9	0.2	
Barriers	0.5	Ba1	0.3	0.6	Barriers score = (Ba1 + (100 − Ba2) + Ba3)/3
		Ba2	0.5	0.3	
		Ba3	0.5	0.5	

Legend: EFA: exploratory factor analysis; item code description provided in Appendix A.

**Table 4:** List of statements used to assess the different Health Belief Model constructs by asking the respondent their degree of agreement through a visual analogue scale.

HBM Construct	Statements Used for the Indirect Assessment of the Constructs
Susceptibility	- In my view, veterinary practitioners are very frequently exposed to zoonotic infectious diseases.
	- In my view, zoonotic infectious diseases represent a major risk for veterinary practitioners.
	- As a veterinary practitioner, I could easily and unwillingly be responsible for the spread of a zoonotic disease to my relatives or to other persons.
	- My future professional practice represents a significant risk to my health.
Severity	- If I were to contract a major zoonotic disease, my income would be heavily impacted.
	- If I were to contract a major zoonotic disease, my life quality would be severely affected.
	- If I were to contract a major zoonotic disease, I might contaminate my relatives and other persons.
Health responsibility	- Veterinary practitioners have an important responsibility towards public health.
	- It is important for veterinary practitioners to respect and apply preventive and control measures while practicing in order to prevent the spread of infectious diseases.
	- Staying healthy is important for both my private and professional life.
Benefits	In your view, what is the efficiency of the following biosecurity measures in preventing your own possible contamination? (0: useless, 100: very effective (full protection))
	- BSM0. The different preventive measures which can be taken by veterinarians.
	- BSM1. Disinfecting hands after each manipulation.
	- BSM2. Asking the owner about the country of origin of the animal in consultation.
	- BSM3. Protecting hands by wearing gloves adapted to the needs.
	- BSM4. Protecting oneself from oro-nasal contaminations by wearing a mask in case of interventions likely to cause projections (e.g., abscess puncture, wound cleaning, descaling, autopsy).
	- BSM5. Protecting oneself against ocular contaminations by wearing protective glasses during interventions likely to cause projections (e.g., descaling, autopsy).
	- BSM6. Throwing needles directly into a specific container without replacing the cap.
	- BSM7. Washing dirty clothing separately with a proper cleaning cycle.
	- BSM8. Being vaccinated against rabies.
	- BSM9. Ensuring proper containment in order to avoid being wounded (bites, scratches, etc.).
	- BSM10. In cases of wounds, proceeding to immediate cleaning with an antiseptic soap or solution.
	- BSM11. Keeping oneself updated on the new developments in terms of zoonosis and their prevention.
	- BSM12. Using a disposable coat a single time.
	- BSM13. Cleaning one’s boots when exiting the holdings.
Barriers	- No measure is really effective; I am exposed to zoonotic infections anyway.
	- Due to my practices, I am able to considerably lower the risks of exposure to and contamination by a zoonotic disease.
	- Undertaking hygienic measures (e.g., hands, boots, etc.) is only possible if the holdings are equipped with proper cleaning infrastructures. If there are no cleaning spots on the holdings, we cannot perform these measures).

Legend: BSM: biosecurity measure.

## Data Availability

Data is contained within the article.

## Appendix A

**Table A1:** Coding and Attributes.

Component	Item Code	Questions or Item Statements	Attributes
Demographic Variables	GI1	Are you male or female?	Male Female
	GI2	Year of studies (students)/year of graduation (practitioners)	Year of education: —2nd year Master’s —3rd year Master’s Year of graduation: - Before 1986 - Between 1986 and 1995 - Between 1996 and 2005 - Between 2006 and 2015 - Between 2016 and now
	GI3	Have you already followed a practitioner or undertaken an internship? (students only)	Yes No
	GI4	If yes, in which kind of practice? (students only)	Large animals Equine Small animals Mixed
	GI5	How many visits or consultations are you doing per day (on average)—rural (practitioners only)	Number
	GI6	How many consultations are you doing per day (on average)—small animals (practitioners only)	Number
Pathways		Classify, in order of importance in terms of zoonotic risks, the following different contamination pathways:	
	PATH1	Aerial	1 to 4
	PATH2	Direct or indirect contact (cutaneous, transcutaneous or oral)	1 to 4
	PATH3	Blood exposure incident (e.g., contaminated needle or blade)	1 to 4
	PATH4	Biological vector	1 to 4
Risk Aversion		Regarding your daily habits (independently of your professional practice), would you agree with the following statements? (0: fully disagree; 100: fully agree)	
	RA1	Compared to others, I consider myself a cautious person.	Visual analogue scale, score from 0 to 100
	RA2	In my life, I usually try to anticipate risks and take specific measures to mitigate them.	
	P1.x	I always bring basic medical products with me for personal use (disinfectant, bandages and pain killers).	
	RA3	I do not usually think of potential incidents; if something happens, we will find a solution at that moment (reversed wording).	
Risk Susceptibility		Do you agree with the following statements? (0: fully disagree; 100: fully agree)	
	Su1	In my view, veterinary practitioners are very frequently exposed to zoonotic infectious diseases.	Visual analogue scale, score from 0 to 100
	Su2	In my view, zoonotic infectious diseases represent a major risk for veterinary practitioners.	
	Su3	As a veterinary practitioner, I could easily and unwillingly be responsible for the spread of a zoonotic disease to my relatives or to other persons.	
	Su4	My future professional practice represents an important risk to my health.	
Risk Severity		Do you agree with the following statements? (0: fully disagree; 100: fully agree)	
	Se1	If I were to contract a major zoonotic disease, my income would be heavily impacted.	Visual analogue scale, score from 0 to 100
	Se2	If I were to contract a major zoonotic disease, my life quality would be severely affected.	
	Se3	If I were to contract a major zoonotic disease, I might contaminate my relatives and other persons.	
Health Responsibility		Would you agree with the following statements? (0: fully disagree; 100: fully agree)	
	HR1	Veterinary practitioners have an important responsibility towards public health.	Visual analogue scale, score from 0 to 100
	HR2	It is important for veterinary practitioners to respect and apply preventive and control measures while practicing to prevent the spread of infectious diseases.	
	HR3	Staying healthy is important for both my private and professional life.	
Benefits of the Biosecurity Measures		In your view, what is the efficiency of the following measures in preventing your own possible contamination? (0: useless; 100: very effective (full protection))	
	Ben_M0	The different preventive measures which can be taken by veterinarians can efficiently reduce the contamination risk for zoonosis (0: No, they are completely useless, 100: Yes, fully effective)	Visual analogue scale, score from 0 to 100
	Ben_M1	Disinfecting hands after each manipulation (or cleaning them with an antibacterial soap or solution)	
	Ben_M2	Asking the owner about the country of origin of the animal in consultation	
	Ben_M3	Protecting one’s hands by wearing gloves adapted to the needs	
	Ben_M4	Protecting oneself from oro-nasal contaminations by wearing a mask in cases of interventions likely to cause projections (e.g., abscess puncture, wound cleaning, descaling, autopsy)	
	Ben_M5	Protecting myself against ocular contaminations by wearing protective glasses during interventions likely to cause projections (e.g., descaling, autopsy)	
	Ben_M6	Throwing needles directly into a specific container without replacing the cap	
	Ben_M7	Washing dirty clothing separately with a proper cleaning cycle	
	Ben_M8	Being vaccinated against rabies	
	Ben_M9	Ensuring proper containment in order to avoid being wounded (bites, scratches, etc.)	
	Ben_M10	In cases of wounds (bites, scratches, etc.), proceeding to immediate cleaning with an antiseptic soap or solution (a few minutes after the event maximum)	
	Ben_M11	Keeping oneself updated on the new developments in terms of zoonosis and their prevention (continuous training)	
	Ben_M12	Using a disposable coat a single time	
	Ben_M13	Cleaning one’s boots when exiting the holdings	
Barriers		Do you agree with the following statements? (0: not at all; 100: fully agree)	
	Ba1	No measure is really effective; I am exposed to zoonotic infections anyway.	Visual analogue scale, score from 0 to 100
	Ba2	Due to my practices, I am able to considerably lower the risks of exposure to and contamination by a zoonotic disease (reversed wording).	
	Ba3	Undertaking hygienic measures (hands, boots, etc.) is only possible if the holdings are equipped with proper cleaning infrastructures. If there are no cleaning spots on the holdings, we cannot perform these measures.	
Intention or Action	BSM1	Do you disinfect your hands after each manipulation (or clean them with an antibacterial soap or solution)?	4: Yes, always 3: Yes, most of the time 2: Yes, sometimes 1: No, never Sub-questions: - If “Yes, most of the time” or “Yes, sometimes”, please indicate in which circumstances you’re planning to implement /will not implement the BSM (if your decision is based on specific criteria) - If “No, never” but you considered this measure as quite efficient, could you inform us as to the main reason you’re not willing to implement it? - If “No, Never”, what could persuade you to implement this measure?
	BSM2	Do you ask the owner about the country of origin of the animal in consultation?	
	BSM3	Do you protect your hands by wearing gloves adapted to the needs?	
	BSM4	Do you protect yourself from oro-nasal contaminations by wearing a mask in case of interventions likely to cause projections (e.g., abscess puncture, wound cleaning, descaling, autopsy)?	
	BSM5	Do you protect yourself against ocular contaminations by wearing protective glasses during interventions likely to cause projections (e.g., descaling, autopsy)?	
	BSM6	Do you throw needles directly into a specific container without replacing the cap?	
	BSM7	Do you wash your dirty work clothing separately with a proper cleaning cycle?	
	BSM8	Did you get vaccinated against rabies before starting your practice?	
	BSM9	Do you ensure proper containment in order to avoid being wounded (bites, scratches, etc.)?	
	BSM10	If you get wounded (bites, scratches, etc.) do you proceed to immediate cleaning with an anti-septic soap or solution?	
	BSM11	Do you get regular updates on new developments in terms of zoonosis and their prevention (continuous training)?	
	BSM12	Do you use disposable coats a single time?	
	BSM13	Do you clean your boots when exiting the holdings?	

## Appendix B

Final Models Obtained through a Backwards Stepwise Analysis Using a Negative Binomial Regression Model and Having at Least One Significant Explanatory Variable.

**Table A2:** Risk aversion, biosecurity knowledge, health responsibility and perceptions of susceptibility, severity and barriers.

Explanatory Variable		Health Responsibility		Susceptibility		Severity		Barriers		Overall Benefits of BSM	
		Estimate	*p*	Estimate	*p*	Estimate	*p*	Estimate	*p*	Estimate	*p*
Respondent (ref = student)	Veterinary practitioner	−0.098	0.000	0.012	0.000	0.087	0.010	0.288	0.000	−0.173	0.000
Gender (ref = Female)	Male					0.062	0.069				
Kind of practice (ref = Large animals)	Mixed										
	Small animals										
Workload											
Risk aversion		0.002	0.000	0.002	0.014	0.002	0.078	−0.008	0.001	0.002	0.000

Legend: *p* is the *p*-value.

**Table A3:** Benefits of the biosecurity measures.

Explanatory Variable		Benefits of BSM0		Benefits of BSM1		Benefits of BSM2		Benefits of BSM3		Benefits of BSM4		Benefits of BSM5			
		Estimate	*p*	Estimate	*p*	Estimate	*p*	Estimate	*p*	Estimate	*p*	Estimate		*p*	
Respondent (ref = student)	Veterinary practitioner	−0.087	0.013			−0.095	0.007								
Gender (ref = Female)	Male			−0.047	0.010	−0.094	0.008			−0.150	0.002				
Kind of practice (ref = Large animals)	Mixed	0.151	0.010	0.098	0.002	0.158	0.011								
	Small animals	0.153	0.006	0.118	0.000	0.220	0.000								
Workload															
Risk aversion		0.002	0.041	0.001	0.010	0.002	0.098	0.003	0.000	0.004	0.005	0.003		0.022	
Explanatory variable		Benefits of BSM7		Benefits of BSM8		Benefits of BSM9		Benefits of BSM10		Benefits of BSM11		Benefits of BSM12		Benefits of BSM13	
		Estimate	*p*	Estimate	*p*	Estimate	*p*	Estimate	*p*	Estimate	*p*	Estimate	*p*	Estimate	*p*
Respondent (ref = student)	Veterinary practitioner	−0.147	0.000	−0.488	0.000										
Gender (ref = Female)	Male														
Kind of practice (ref = Large animals)	Mixed														
	Small animals														
Workload						−0.006	0.002								
Risk aversion	Psych1	0.003	0.025			0.003	0.021	0.001	0.044	0.001	0.026	0.005	0.023	0.001	0.018

Legend: *p* is the *p*-value.

**Table A4:** Intention or implementation of the biosecurity measure.

Explanatory Variable		M1		M2		M3		M5		M6		M7	
		Estimate	*p*	Estimate	*p*	Estimate	*p*	Estimate	*p*	Estimate	*p*	Estimate	*p*
Perceptions based on the Health Belief model	Susceptibility												
	Severity												
	Health responsibility												
	Benefit measure	0.006	0.010	0.007	0.000	0.004	0.020	0.010	0.000	0.005	0.000	0.006	0.000
		M8		M10		M11		M12		Overall BS Score			
		Estimate	*p*	Estimate	*p*	Estimate	*p*	Estimate	*p*	Estimate	*p*		
Perceptions based on the Health Belief model	Susceptibility									0.001	0.015		
	Severity									−0.001	0.000		
	Health responsibility	0.006	0.060										
	Benefit measure	0.004	0.001	0.004	0.058	0.006	0.003	0.008	0.003	0.009	0.000		

Legend: *p* is the *p*-value.

## Appendix C

**Questionnaire for Veterinary Practitioners**

**Perception survey on zoonotic risks and preventive measures in veterinary practices**

This questionnaire has been launched by the University of Liege as part of a research project aiming at analysing perceptions of veterinary students regarding zoonotic risks and the efficiency of the different measures recommended to prevent zoonotic infections.

It is addressed to all veterinary practitioners.

This survey is anonymous and takes around 15 minutes to complete. It can be saved and finalised later if necessary.

There are 84 questions in this survey

**General Information**

**Are you a male or female?**

Female     Male

**In which year did you obtain your veterinary degree?**

Please choose **only one** of the following:

Between 2016 and now  Between 2006 and 2015

Between 1996 and 2005

Between 1986 and 1995   Before 1986

**What type of practice do you have?** Please choose **only one** of the following:

Large animals/Equine   Small animals   Mixed   Other

**How many visits do you make per day (on average)?**

**Only answer this question if the following conditions are met:** Answer was “Large animals/Equine” at question “4 [D3]” (What type of practice do you have?)

Your answer must be between 0 and 99, only an integer value may be entered in this field.

**How many consultations d you undertake per day (on average)?**

**Only answer this question if the following conditions are met:** *Answer was “Small animals” at question “4 [D3]” (What type of practice do you have?)*

Your answer must be between 0 and 99, only an integer value may be entered in this field.

**On average and per day, how many visits and consultations do you undertake?**

***Only answer this question if the following conditions are met:*** *Answer was “Mixed” or “Other” at question “4 [D3]” (What type of practice do you have?)*

Visits: Consultations for small animals:

**Profile**

**Regarding your daily habits (independently of your professional practice), would you agree with the following statements? (0: fully disagree; 100: fully agree)**

- Compared to others, I consider myself a cautious person.
- In my life, I usually try to anticipate risks and take specific measures to mitigate them.
- I do not usually think of possible incidents. If something happens, we will find a solution at that moment.

**Would you agree with the following statements? (0: fully disagree; 100: fully agree)**

- As veterinary practitioner, I have an important responsibility towards public health.
- Applying preventive and control measures for infectious diseases while practicing is essential to prevent their dissemination.
- Staying healthy is important for both my private and professional life.

**Risk and Severity**

**Do you agree with the following statements?** (0: not at all; 100: fully agree)

- In my view, veterinary practitioners are very frequently exposed to zoonotic infectious diseases.
- In my view, zoonotic infectious diseases represent a major risk for veterinary practitioners.
- As a veterinary practitioner, I could easily and unwillingly be responsible for the spread of a zoonotic disease to my relatives or to other persons.
- My professional practice represents an important risk for my health.

**Have you been personally affected by a zoonotic disease in the past?**

Yes     No

**What was the impact of the disease on your health or income?** (0: none, 100: very important (e.g., long term invalidity with incapacity to work))

**Only answer this question if the following conditions are met:** Answer was “Yes” at question “11 [Su2]” (Have you been personally affected by a zoonotic disease in the past?)

**Among persons you know, have any veterinarians been affected by a zoonotic disease in the past?**

Yes     No

**What was the impact of the disease on their health and/or income?** (0: none, 100: very important (e.g., long-term invalidity with incapacity for work))

***Only answer this question if the following conditions are met:*** *Answer was “Yes” at question “13 [Su3]” (Among persons you know, have any veterinarians been affected by a zoonotic disease in the past?)*

**Classify, by order of importance in terms of zoonotic risks, the different contamination pathways.** *(1 = the more important; 4: the less important)

- Aerial
- Direct or indirect contact (cutaneous, transcutaneous or oral)
- Blood exposure (e.g., blades or contaminated needles)
- Biological vector

**Do you agree with the following statements?** (0: not at all, 100: fully agree)

- If I were to contract a major zoonotic disease, my income would be heavily impacted.
- If I were to contract a major zoonotic disease, my life quality would be severely affected.
- The majority of the zoonotic diseases we are regularly exposed to have no or few consequences on our health or our relatives’ health.
- If I were to contract a major zoonotic disease, I might contaminate my relatives and other persons.

**Do you consider that the different preventive measures which can be taken by veterinarians can efficiently reduce the contamination risk for zoonosis? (0: No, they are useless; 100: Yes, fully effective)**

**Efficiency of Prevention and Control Measures**

In your view, what is the efficiency of the following measures in prevent your own possible contamination? *(0: useless, 100: very effective (full protection))*

1. Disinfecting hands after each manipulation (or cleaning them with an antibacterial soap or solution). *
2. Asking the owner about the country of origin of the animal in consultation. *
3. Protecting one’s hands by wearing gloves adapted to the needs. *
4. Protecting oneself from oro-nasal contaminations by wearing a mask in cases of interventions likely to cause projections (e.g., abscess puncture, wound cleaning, descaling, autopsy, etc.). *
5. Protecting oneself against ocular contaminations by wearing protective glasses during interventions likely to cause projections (e.g., descaling, autopsy, etc.). *
6. Not recapping needles (throwing them directly into a specific container). *
7. Washing dirty clothing separately with a proper cleaning cycle. *
8. Being vaccinated against rabies. *
9. Ensuring proper containment in order to avoid being wounded (bites, scratches, etc.). *
10. In cases of wounds (bites, scratches, etc.), proceeding to immediate cleaning with an antiseptic soap or solution (a few minutes after the event maximum). *
11. Keeping oneself updated of new developments in terms of zoonosis and their prevention (continuous training). *
12. Using a disposable coat a single time. *

***Only answer this question if the following conditions are met:*** *Answer was “Large animals/Equine” or “Mixed” at question “4 [D3]” (What type of practice do you have?)*

13. Cleaning my boots when exiting the holdings. *

***Only answer this question if the following conditions are met:*** *Answer was “Large animals/Equine” or “Mixed” at question “4 [D3]” (What type of practice do you have?)*

**Possible Constraints**

**Do you agree with the following statements? (0: not at all, 100: fully agree) ***

- No measure is really effective; I am exposed to zoonotic infections anyway.
- Due to my practices, I am able to considerably lower the risks of exposure to and contamination by a zoonotic disease.
- Undertaking hygienic measures (hands, boots, etc.) is only possible if the holdings are equipped with proper cleaning infrastructures. If there are no cleaning spots on the holdings we cannot perform these measures.

**Implementation**

1. **Do you disinfect your hands after each manipulation (or clean them with an antibacterial soap or solution)?**

Yes, always   Yes, most of the time   Yes, sometimes   No, never

**→ If you are doing it but not always, please clarify in which circumstances you are doing or not doing it?**

**→ You considered this measure as quite efficient but you’re not implementing it. Could you inform us as to the main reason for not doing it?**

***Only answer this question if the answer was*** *“No, never” and the benefits were rated at 50 or more*

Too expensive   Takes too long   Not feasible in practice

Not well perceived by clients   Other

**→ If “No, never”, what could persuade you to implement this measure?**

2. **Do you ask the owner about the country of origin of the animal in consultation? ***

Yes, always   Yes, most of the time     Yes, sometimes   No, never

**→ If you are doing it but not always, please clarify in which circumstances you are doing or not doing it?**

**→ You considered this measure as quite efficient but you’re not implementing it. Could you inform us as to the main reason for not doing it?**

***Only answer this question if the answer was*** *“No, never” and the benefits were rated at 50 or more*

Too expensive   Takes too long   Not feasible in practice

Not well perceived by clients   Other

**→ If “No, never”, what could persuade you to implement this measure?**

3. **Do you protect your hands by wearing gloves adapted to the needs?**

Yes, always   Yes, most of the time   Yes, sometimes   No, never

**→ If you are doing it but not always, please clarify in which circumstances you are doing or not doing it?**

**→ You considered this measure as quite efficient but you’re not implementing it. Could you inform us as to the main reason for not doing it?**

***Only answer this question if the answer was*** *“No, never” and the benefits were rated at 50 or more*

Too expensive   Takes too long   Not feasible in practice

Not well perceived by clients   Other

**→ If “No, never”, what could persuade you to implement this measure?**

4. **Do you protect yourself from oro-nasal contaminations by wearing a mask in cases of interventions likely to cause projections (e.g., abscess puncture, wound cleaning, descaling, autopsy, etc.)?**

Yes, always   Yes, most of the time    Yes, sometimes   No, never

**→ If you are doing it but not always, please clarify in which circumstances you are doing or not doing it?**

**→ You considered this measure as quite efficient but you’re not implementing it. Could you inform us as to the main reason for not doing it?**

***Only answer this question if the answer was*** *“No, never” and the benefits were rated at 50 or more*

Too expensive   Takes too long   Not feasible in practice

Not well perceived by clients   Other

**→ If “No, never”, what could persuade you to implement this measure?**

5. **Do you protect yourself against ocular contaminations by wearing protective glasses during interventions likely to cause projections (e.g., descaling, autopsy, etc.)?**

Yes, always   Yes, most of the time   Yes, sometimes   No, never

**→ If you are doing it but not always, please clarify in which circumstances you are doing or not doing it?**

**→ You considered this measure as quite efficient but you’re not implementing it. Could you inform us as to the main reason for not doing it?**

***Only answer this question if the answer was*** *“No, never” and the benefits were rated at 50 or more*

Too expensive   Takes too long   Not feasible in practice

Not well perceived by clients   Other

**→ If “No, never”, what could persuade you to implement this measure?**

6. **Do you throw the needles directly into a specific container without recapping them?**

Yes, always   Yes, most of the time     Yes, sometimes   No, never

**→ If you are doing it but not always, please clarify in which circumstances you are doing or not doing it?**

**→ You considered this measure as quite efficient but you’re not implementing it. Could you inform us as to the main reason for not doing it?**

***Only answer this question if the answer was*** *“No, never” and the benefits were rated at 50 or more*

Too expensive   Takes too long   Not feasible in practice

Not well perceived by clients   Other

**→ If “No, never”, what could persuade you to implement this measure?**

7. **Do you wash your dirty work clothing separately with a proper cleaning cycle?**

Yes, always   Yes, most of the time     Yes, sometimes   No, never

**→ If you are doing it but not always, please clarify in which circumstances you are doing or not doing it?**

**→ You considered this measure as quite efficient but you’re not implementing it. Could you inform us as to the main reason for not doing it?**

***Only answer this question if the answer was*** *“No, never” and the benefits were rated at 50 or more*

Too expensive   Takes too long   Not feasible in practice

Not well perceived by clients   Other

**→ If “No, never”, what could persuade you to implement this measure?**

8. **Are you vaccinated against rabies?**

Yes, I am vaccinated and up to date.

Yes, I am vaccinated but I have not followed the recommendations regarding the renewal of the vaccine.

No, I have never been vaccinated against rabies.

**→ If you have been vaccinated in the past but have not been revaccinated in accordance with the recommendations, could you tell us why?**

**→ You considered this measure as quite efficient but you’re not implementing it. Could you inform us as to the main reason for not doing it?**

***Only answer this question if the answer was*** *“No, never” and the benefits were rated at 50 or more*

Too expensive   Takes too long   Not feasible in practice

Not well perceived by clients   Other

**→ If “No, never”, what could persuade you to implement this measure?**

9. **Do you ensure proper containment in order to avoid being wounded (bites, scratches, etc.)?**

Yes, always   Yes, most of the time     Yes, sometimes   No, never

**→ If you are doing it but not always, please clarify in which circumstances you are doing or not doing it?**

**→ You considered this measure as quite efficient but you’re not implementing it. Could you inform us as to the main reason for not doing it?**

***Only answer this question if the answer was*** *“No, never” and the benefits were rated at 50 or more*

Too expensive   Takes too long   Not feasible in practice

Not well perceived by clients   Other

**→ If “No, never”, what could persuade you to implement this measure?**

10. **If you get wounded (bites, scratches, etc.), do you proceed to immediate cleaning with an antiseptic soap or solution (a few minutes after the event maximum)?**

Yes, always   Yes, most of the time   Yes, sometimes   No, never

**→ If you are doing it but not always, please clarify in which circumstances you are doing or not doing it?**

**→ You considered this measure as quite efficient but you’re not implementing it. Could you inform us as to the main reason for not doing it?**

***Only answer this question if the answer was*** *“No, never” and the benefits were rated at 50 or more*

Too expensive   Takes too long   Not feasible in practice

Not well perceived by clients   Other

**→ If “No, never”, what could persuade you to implement this measure?**

11. **Do you get regular updates on new developments in terms of zoonosis and their prevention (continuous training)?**

Yes, always   Yes, most of the time   Yes, sometimes   No, never

**→ If you are doing it but not always, please clarify in which circumstances you are doing or not doing it?**

**→ You considered this measure as quite efficient but you’re not implementing it. Could you inform us as to the main reason for not doing it?**

***Only answer this question if the answer was*** *“No, never” and the benefits were rated at 50 or more*

Too expensive   Takes too long   Not feasible in practice

Not well perceived by clients   Other

**→ If “No, never”, what could persuade you to implement this measure?**

12. **Do you use disposable coats a single time?**

***Only answer this question if the following conditions are met:*** *Answer was “Large animals/Equine” or “Mixed” at question “4 [D3]” (What type of practice do you have?)*

Yes, always   Yes, most of the time   Yes, sometimes   No, never

**→ If you are doing it but not always, please clarify in which circumstances you are doing or not doing it?**

**→ You considered this measure as quite efficient but you’re not implementing it. Could you inform us as to the main reason for not doing it?**

***Only answer this question if the answer was*** *“No, never” and the benefits were rated at 50 or more*

Too expensive   Takes too long   Not feasible in practice

Not well perceived by clients   Other

**→ If “No, never”, what could persuade you to implement this measure?**

13. **Do you clean your boots when exiting the holdings?**

***Only answer this question if the following conditions are met:*** *Answer was “Large animals/Equine” or “Mixed” at question “4 [D3]” (What type of practice do you have?)*

Yes, always   Yes, most of the time   Yes, sometimes   No, never

**→ If you are doing it but not always, please clarify in which circumstances you are doing or not doing it?**

**→ You considered this measure as quite efficient but you’re not implementing it. Could you inform us as to the main reason for not doing it?**

***Only answer this question if the answer was*** *“No, never” and the benefits were rated at 50 or more*

Too expensive   Takes too long   Not feasible in practice

Not well perceived by clients   Other

**→ If “No, never” what could persuade you to implement this measure?**

**Comment**

**Do you have any additional comments or suggestions?**

Thank you for your participation.

If you have any questions regarding this survey or the research project, feel free to contact Véronique Renault at the following e-mail address: vrenault@uliege.be.

22 February 2021—14:34

Submit your survey.

Thank you for completing this survey.

## Appendix D

**Questionnaire for veterinary students**

**Perception survey on zoonotic risks and preventive measures in veterinary practices**

This questionnaire has been launched by the University of Liege as part of a research project aiming at analysing the perceptions of veterinary students regarding zoonotic risks and the efficiency of the different measures recommended to prevent zoonotic infections.

It is addressed to veterinary students in the final years of study.This survey is anonymous and takes around 15 minutes to complete. It can be saved and finalised later if necessary.

There are 85 questions in this survey

**General Information**

**Are you a male or female?**

Female    Male

**In which year of studies are you?**

2nd year Master’s   3rd year Master’s

**Is one of your close relatives a veterinary practitioner?**

Yes    No

**→ If yes:—What is your relationship?**

*If you have a family tie with several veterinary practitioners, please mention the closest relative.*

**What type of practice do they have?**

Large animals/Equine   Small animals   Mixed   Other

**Have you already followed a practitioner or undertaken an internship?**

Yes    No

**→ If yes, in which kind of practices?**

Large animals/Equine   Small animals   Mixed   Other

**Profile**

**Regarding your daily habits (independently of your professional practice), would you agree with the following statements? (0: fully disagree; 100: fully agree)**

- Compared to others, I consider myself a cautious person.
- In my life, I usually try to anticipate risks and take specific measures to mitigate them.
- I do not usually think of possible incidents. If something happens, we will find a solution at that moment.

**Would you agree with the following statements? (0: fully disagree; 100: fully agree)**

- Veterinary practitioners have an important responsibility towards public health.
- It is important for veterinary practitioners to respect and apply preventive and control measures while practicing to prevent the spread of infectious diseases.
- Staying healthy is important for both my private and professional life.

**Risk and Severity**

**Do you agree with the following statements? (0: not at all; 100: fully agree)**

- In my view, veterinary practitioners are very frequently exposed to zoonotic infectious diseases.
- In my view, zoonotic infectious diseases represent a major risk for veterinary practitioners.
- As a veterinary practitioner, I could easily and unwillingly be responsible for the spread of a zoonotic disease to my relatives or to other persons.
- My professional practice represents an important risk to my health.

**Have you been personally affected by a zoonotic disease in the past?**

Yes   No

**What was the impact of the disease on your health or income?** (0: none; 100: very important (e.g., long term invalidity with incapacity to work))

***Only answer this question if the following conditions are met:*** *Answer was “Yes” at question “11 [Su2]” (Have you been personally affected by a zoonotic disease in the past?)*

**Among persons you know, have any veterinarians been affected by a zoonotic disease in the past?**

Yes    No

**What was the impact of the disease on their health and/or income?** (0: none; 100: very important (e.g., long-term invalidity with incapacity to work))

***Only answer this question if the following conditions are met:*** *Answer was “Yes” at question “13 [Su3]” (Among persons you know, have any veterinarians been affected by a zoonotic disease in the past?)*

**Classify, by order of importance in terms of zoonotic risks, the different contamination pathways. ***(1: more important; 4: less important)

- Aerial
- Direct or indirect contact (cutaneous, transcutaneous or oral)
- Blood exposure (e.g., blades or contaminated needles)
- Biological vector

**Do you agree with the following statements?**

- As a veterinary practitioner, if I were to contract a major zoonotic disease, my income would be heavily impacted.
- As a veterinary practitioner, if I were to contract a major zoonotic disease, my life quality would be severely affected.
- The majority of the zoonotic diseases likely to affect veterinarians have no or few consequences on their health or their relatives’ health.
- If I were to contract a major zoonotic disease, I might contaminate my relatives and other persons.

**Do you consider that the different preventive measures which can be taken by veterinarians can efficiently reduce the contamination risk for zoonosis? (0: No, they are useless; 100: Yes, fully effective)**

**Efficiency of Prevention and Control Measures**

According to you, what is the efficiency of the following measures to prevent your own possible contamination *(0: useless; 100: very effective (full protection))*

1. Disinfecting hands after each manipulation (or cleaning them with an antibacterial soap or solution). *
2. Asking the owner about the country of origin of the animal in consultation. *
3. Protecting one’s hands by wearing gloves adapted to the needs. *
4. Protecting oneself from oro-nasal contaminations by wearing a mask in case of interventions likely to cause projections (e.g., abscess puncture, wound cleaning, descaling, autopsy, etc.). *
5. Protecting oneself against ocular contaminations by wearing protective glasses during interventions likely to cause projections (e.g., descaling, autopsy, etc.). *
6. Not recapping needles (throwing them directly into a specific container). *
7. Washing dirty clothing separately with a proper cleaning cycle. *
8. Being vaccinated against rabies. *
9. Ensuring proper containment in order to avoid being wounded (bites, scratches, etc.). *
10. In cases of wound (bites, scratches, etc.), proceeding to immediate cleaning with an antiseptic soap or solution (a few minutes after the event maximum). *
11. Keeping myself updated of new developments in terms of zoonosis and their prevention (continuous training). *
12. Using a disposable coat a single time. *
13. Cleaning my boots when exiting the holdings. *

**Possible constraints**

**Do you agree with the following statements? (0: not at all; 100: fully agree) ***

- No measure is really effective, I am exposed to zoonotic infections anyway.
- Due to my practices, I am able to considerably lower the risks of exposure to and contamination by a zoonotic disease.
- Undertaking hygienic measures (hands, boots, etc.) is only possible if the holdings are equipped with proper cleaning infrastructures. If there are no cleaning spots on the holdings we cannot perform these measures

**Implementation**

When you are a veterinary practitioner, do you intend to implement the following measures?

**1. Do you disinfect your hands after each manipulation (or clean them with an antibacterial soap or solution)?**
   Yes, always   Yes, most of the time    Yes, sometimes   No, never
  **→ If you are doing it but not always, please clarify in which circumstances you are doing or not doing it?**
  **→ You considered this measure as quite efficient but you’re not implementing it. Could you inform as to the main reason for not doing it?**
  ***Only answer this question if the answer was*** *“No, never” and the benefits were rated at 50 or more*
   Too expensive   Takes too long   Not feasible in practice
   Not well perceived by clients   Other
  **→ If “No, never”, what could persuade you to implement this measure?**

**2. Do you ask the owner about the country of origin of the animal in consultation? ***
   Yes, always   Yes, most of the time   Yes, sometimes   No, never
  **→ If you are doing it but not always, please clarify in which circumstances you are doing or not doing it?**
  **→ You considered this measure as quite efficient but you’re not implementing it. Could you inform us as to the main reason for not doing it?**
  ***Only answer this question if the answer was*** *“No, never” and the benefits were rated at 50 or more*
   Too expensive   Takes too long   Not feasible in practice
   Not well perceived by clients   Other
  **→ If “No, never”, what could persuade you to implement this measure?**

**3. Do you protect your hands by wearing gloves adapted to the needs?**
   Yes, always   Yes, most of the time     Yes, sometimes   No, never
  **→ If you are doing it but not always, please clarify in which circumstances you are doing or not doing it?**
  **→ You considered this measure as quite efficient but you’re not implementing it. Could you inform us as to the main reason for not doing it?**
  ***Only answer this question if the answer was*** *“No, never” and the benefits were rated at 50 or more*
   Too expensive   Takes too long   Not feasible in practice
   Not well perceived by clients   Other
  **→ If “No, never”, what could persuade you to implement this measure?**

**4. Do you protect yourself from oro-nasal contaminations by wearing a mask in cases of interventions likely to cause projections (e.g., abscess puncture, wound cleaning, descaling, autopsy, etc.)?**
   Yes, always   Yes, most of the time     Yes, sometimes   No, never
  **→ If you are doing it but not always, please clarify in which circumstances you are doing or not doing it?**
  **→ You considered this measure as quite efficient but you’re not implementing it. Could you inform us as to the main reason for not doing it?**
  ***Only answer this question if the answer was*** *“No, never” and the benefits were rated at 50 or more*
   Too expensive   Takes too long   Not feasible in practice
   Not well perceived by clients   Other
  **→ If “No, never”, what could persuade you to implement this measure?**

**5. Do you protect yourself against ocular contaminations by wearing protective glasses during interventions likely to cause projections (e.g., descaling, autopsy, etc.)?**
   Yes, always   Yes, most of the time     Yes, sometimes   No, never
  **→ If you are doing it but not always, please clarify in which circumstances you are doing or not doing it?**
  **→ You considered this measure as quite efficient but you’re not implementing it. Could you inform us as to the main reason for not doing it?**
  ***Only answer this question if the answer was*** *“No, never” and the benefits were rated at 50 or more*
   Too expensive   Takes too long   Not feasible in practice
   Not well perceived by clients   Other
  **→ If “No, never”, what could persuade you to implement this measure?**

**6. Do you throw needles directly into a specific container without recapping them?**
   Yes, always   Yes, most of the time     Yes, sometimes   No, never
  **→ If you are doing it but not always, please clarify in which circumstances you are doing it or not doing it?**
  **→ You considered this measure as quite efficient but you’re not implementing it. Could you inform us on the main reason for not doing it?**
  ***Only answer this question if the answer was*** *“No, never” and the benefits were rated at 50 or more*
   Too expensive   Takes too long   Not feasible in practice
   Not well perceived by clients   Other
  **→ If “No, never”, what could persuade you to implement this measure?**

**7. Do you wash your dirty work clothing separately with a proper cleaning cycle?**
   Yes, always   Yes, most of the time   Yes, sometimes   No, never
  **→ If you are doing it but not always, please clarify in which circumstances you are doing or not doing it?**
  **→ You considered this measure as quite efficient but you’re not implementing it. Could you inform us as to the main reason for not doing it?**
  ***Only answer this question if the answer was*** *“No, never” and the benefits were rated at 50 or more*
   Too expensive   Takes too long   Not feasible in practice
   Not well perceived by clients   Other
  **→ If “No, never”, what could persuade you to implement this measure?**

**8. Are you vaccinated against rabies?**
   Yes, I am vaccinated and up to date.
   Yes, I am vaccinated but I have not followed the recommendations regarding the renewal of the vaccine.
   No, I have never been vaccinated against rabies.
  **→ If you have been vaccinated in the past but have not been revaccinated in accordance with the recommendations, could you tell us why ?**
  **→ You considered this measure as quite efficient but you’re not implementing it. Could you inform us as to the main reason for not doing it?**
  ***Only answer this question if the answer was*** *“No, never” and the benefits were rated at 50 or more*
   Too expensive   Takes too long   Not feasible in practice
   Not well perceived by clients   Other
  **→ If “No, never”, what could persuade you to implement this measure?**

**9. Do you ensure proper containment in order to avoid being wounded (bites, scratches, etc.)?**
   Yes, always   Yes, most of the time   Yes, sometimes   No, never
  **→ If you are doing it but not always, please clarify in which circumstances you are doing or not doing it?**
  **→ You considered this measure as quite efficient but you’re not implementing it. Could you inform us as to the main reason for not doing it?**
  ***Only answer this question if the answer was*** *“No, never” and the benefits were rated at 50 or more*
   Too expensive   Takes too long   Not feasible in practice
   Not well perceived by clients   Other
  **→ If “No, never”, what could persuade you to implement this measure?**

**10. If you get wounded (bites, scratches, etc.), do you proceed to immediate cleaning with an antiseptic soap or solution (a few minutes after the event maximum)?**
   Yes, always   Yes, most of the time   Yes, sometimes   No, never
  **→ If you are doing it but not always, please clarify in which circumstances you are doing or not doing it?**
  **→ You considered this measure as quite efficient but you’re not implementing it. Could you inform us as to the main reason for not doing it?**
  ***Only answer this question if the answer was*** *“No, never” and the benefits were rated at 50 or more*
   Too expensive   Takes too long   Not feasible in practice
   Not well perceived by clients   Other
  **→ If “No, never”, what could persuade you to implement this measure?**

**11. Do you get regular updates on new developments in terms of zoonosis and their prevention (continuous training)?**
   Yes, always   Yes, most of the time   Yes, sometimes   No, never
  **→ If you are doing it but not always, please clarify in which circumstances you are doing or not doing it?**
  **→ You considered this measure as quite efficient but you’re not implementing it. Could you inform us as to the main reason for not doing it?**
  ***Only answer this question if the answer was*** *“No, never” and the benefits were rated at 50 or more*
   Too expensive   Takes too long   Not feasible in practice
   Not well perceived by clients   Other
  **→ If “No, never”, what could persuade you to implement this measure?**

**12. Do you use disposable coats a single time?**
   Yes, always   Yes, most of the time   Yes, sometimes   No, never
  **→ If you are doing it but not always, please clarify in which circumstances you are doing or not doing it?**
  **→ You considered this measure as quite efficient but you’re not implementing it. Could you inform us as to the main reason for not doing it?**
  ***Only answer this question if the answer was*** *“No, never” and the benefits were rated at 50 or more*
   Too expensive   Takes too long   Not feasible in practice
   Not well perceived by clients   Other
  **→ If “No, never”, what could persuade you to implement this measure?**

**13. Do you clean your boots when exiting the holdings?**
   Yes, always   Yes, most of the time   Yes, sometimes   No, never
  **→ If you are doing it but not always, please clarify in which circumstances you are doing or not doing it?**
  **→ You considered this measure as quite efficient but you’re not implementing it. Could you inform us as to the main reason for not doing it?**
  ***Only answer this question if the answer was*** *“No, never” and the benefits were rated at 50 or more*
   Too expensive   Takes too long   Not feasible in practice
   Not well perceived by clients   Other
  **→ If “No, never” what could persuade you to implement this measure?**

**Comment**

Do you have any additional comments or suggestions?

Thank you for your participation.

If you have any questions regarding this survey or the research project, feel free to contact Véronique Renault at the following e-mail address: vrenault@uliege.be.

22 February 2021—14:34

Submit your survey.

Thank you for completing this survey.
